# Conclusion

**DOI:** 10.1038/sj.bjc.6601480

**Published:** 2003-12-10

**Authors:** H Cortes-Funes

**Affiliations:** 1 Hospital Universitario 12 de Octubre, Avda Córdoba Km 5,4, Madrid 28041, Spain

The ‘Iressa’ Clinical Experience (ICE) meeting in Madrid provided a unique opportunity for physicians to discuss their real-life experiences with gefitinib (‘Iressa’, ZD1839), and has highlighted the scope and potential of gefitinib therapy in patients with non-small-cell lung cancer (NSCLC). The diverse patient population in the Expanded Access Programme (EAP) included chemonaive to eighth-line patients, patients with performance status 0–4 or comorbidity, and elderly patients. Clinical efficacy and safety data from these EAP patients provide further evidence that gefitinib is active and well tolerated, supporting results from the IDEAL (‘Iressa’ Dose Evaluation in Advanced Lung cancer) clinical trials, which used more defined patient populations (Fukuoka *et al*, 2003; Kris *et al*, 2003).

The diversity of case reports/series presented at the ICE meeting has identified many potential areas of interest for physicians. For example, preliminary data from the EAP support further investigations regarding the efficacy of gefitinib in elderly patients and patients with brain metastases, as well as the sequencing of chemotherapy after gefitinib. In addition, trials in patients with poor performance, patients who are at high risk, patients with bronchioalveolar carcinoma and chemonaive patients are currently ongoing or in the planning stages.

Future development of gefitinib also includes AstraZeneca-sponsored Phase III trials in NSCLC, with two studies of gefitinib 250 mg day^−1^ plus best supportive care (BSC) compared with placebo plus BSC in previously treated patients. Trial 709, also known as ISEL (‘Iressa’ Survival Evaluation in Lung cancer), involves patients who have received platinum and docetaxel, with the primary end point being superior survival. The second, trial 710 or IBREESE (‘Iressa’ *vs* BSC: a Randomised Evaluation of Effect on pulmonary Symptom Endpoint), has pulmonary symptom improvement rate as the primary end point. Gefitinib 250 mg day^−1^ is being compared with docetaxel (75 mg m^−2^ every 3 weeks) as second-line treatment in a randomised open-label trial (721), with co-primary end points of time to progression and survival, and additional studies will assess the potential for gefitinib in the first-line setting. A range of other investigations in NSCLC include cooperative group trials, evaluating gefitinib maintenance after chemoradiation and adjuvant gefitinib after resection, and an AstraZeneca Investigator Initiated Trial programme.

Together with the identification of molecular and genetic markers for sensitivity and resistance to gefitinib, these trials will enable a more precise identification of which patients should receive gefitinib, when they should receive it for optimum benefit and if and how it should be sequenced with chemotherapy. Using this rational approach, we will be able to maximise the benefit of this novel agent for patients with NSCLC.

This meeting successfully met the objectives of bringing together physicians from across the world to share and learn from each other's clinical experience of gefitinib, and has raised a number of questions for consideration. These questions will be addressed by an ongoing comprehensive clinical trial programme, with the ultimate aim of improving patient care.
